# Fruit and Vegetable Intake, Food Security, Barriers to Healthy Eating, and Empowerment among Dietetic Interns and Physician Assistant Interns: A Cross-Sectional Pilot Study

**DOI:** 10.3390/nu16071034

**Published:** 2024-04-02

**Authors:** Alison Campbell, Maren Stirling, Natalie Keele, Alisse Larkin, Rachel Crandall, Aubree N. Wilcox, Meghan Adair, Cloie Malan, Jaidyn Thomson, Alexis Bennett, Heather Jensen, Hadyn Flygare, Lacie Peterson, Holly Hopkins, Nikki Kendrick, Maria Givler, Rebecca Charlton, Katie N. Kraus

**Affiliations:** Department of Nutrition, Dietetics & Food Sciences, College of Agricultural and Applied Sciences, Utah State University, Logan, UT 84322, USA; alison.campbell@usu.edu (A.C.); a02249676@usu.edu (M.S.); a02255619@usu.edu (N.K.); a02341537@usu.edu (A.L.); a02275586@usu.edu (R.C.); a02273895@usu.edu (A.N.W.); a02220227@usu.edu (M.A.); a02285330@usu.edu (C.M.); a02220400@usu.edu (J.T.); a02252434@usu.edu (A.B.); a02317180@usu.edu (H.J.); a02314490@usu.edu (H.F.); lacie.peterson@usu.edu (L.P.); holly.hopkins@usu.edu (H.H.); nikki.kendrick@usu.edu (N.K.); maria.givler@usu.edu (M.G.); rebecca.charlton@usu.edu (R.C.)

**Keywords:** food security, physician assistant, registered dietitian, dietetics, student, intern, empowerment, nutrition, preceptor, supervised practice

## Abstract

Students are required to complete supervised practice hours prior to becoming Registered Dietitians and Physician Assistants. Research suggests that environmental and social factors affect dietetic interns’ diets during their internship, although these factors have not been studied among physician assistant interns. This cross-sectional study utilized an online survey to compare dietetic interns’ (*n* = 81) and physician assistant interns’ (*n* = 79) fruit and vegetable intake, food security, barriers to healthy eating, and empowerment for making healthy dietary choices during an internship. Differences were assessed via independent *t*-tests and chi-square distributions. The significance was set at *p* < 0.05. Dietetic interns had a higher vegetable intake (*p* = 0.002) while physician assistant interns had higher rates of food insecurity (*p* = 0.040). Dietetic interns reported a greater impact on their dietary choices due to mental fatigue (*p* = 0.006), while physician assistant interns’ dietary choices were more heavily impacted by peer influence, interactions with patients, and interactions with preceptors (*p* < 0.05). There was not a group difference in overall empowerment (*p* = 0.157), although both groups rated empowerment for asking for help with food and nutrition challenges the lowest of the empowerment sub-items. Addressing interns’ unique needs may support students’ educational success and wellbeing once they are professionals, promote a diverse workforce, and ensure optimal care for patients.

## 1. Introduction

Healthcare provider internships are the foundational training and primary way that students develop future practices as healthcare providers. For many students, this is a challenging portion of their education as it may require relocation, becoming familiar with one or many preceptors and facilities, and often follows the schedule of a full-time job [1]. Rigorous training and internship programs may not support the healthy behaviors that interns aspire to instill in their patients or clients. A decline in healthy habits and decreased access and intake of healthy foods may contribute to intern burnout [2], poor mental health, and poor academic outcomes such as impaired concentration and a lower grade point average [3]. These outcomes may negatively impact the care that is provided to patients or clients due to a lack of empathy [4] and increased medical errors [5]. Recent research on the topics of healthcare provider burnout have shown that nutrition and other lifestyle behaviors can have a significant impact on overall health and workplace engagement [6,7,8]. Our healthcare system has a higher reliance on allied health professionals and it is critical that they remain in the workplace.

College student diets often do not align with dietary recommendations. According to the Spring 2023 American College Health Association National College Health Assessment (ACHA-NCHA), 17% of students reported eating three servings or more of fruit, and 29% reported eating three or more servings of vegetables daily [9]. A small study of dietetic students in Turkey reported an average intake of 1.96 ± 0.19 servings of fruit daily, and 2.91 ± 0.08 of vegetables daily, both of which were higher than college students who were not studying nutrition [10]. Fruit and vegetable intake has not been assessed among physician assistant interns. The ACHA-NCHA report also indicated that 46.6% of college students had some level of food insecurity [9]. More research is needed on the prevalence of food security among dietetic interns or physician assistant interns. Other research suggests that time, finances, food accessibility, and physical, mental, and social health influence dietetic interns’ diets during their internship [1]. However, research assessing barriers to healthy eating among physician assistant interns has not been explicitly addressed.

Some interns may feel that their dietary habits are out of their control due to the circumstances of their internship. Although their situations may not be optimal, there are often decisions they can make to benefit their health. Previous research suggests that individual empowerment, or the perception and belief that an individual has control and responsibility for their quality of life [10,11,12], is correlated with healthy behaviors including exercise and following a healthy diet [11]. Interns’ empowerment for healthy eating during an internship has not previously been studied.

A lack of healthy behaviors during internship training could lead to long-term practices that may cause burnout sooner. Although research exists regarding many of these factors among college students in general, more research is needed to elucidate the prevalence and impact of these factors on dietetic interns and physician assistant interns. We hypothesize that healthy eating during training could create healthy habits that may prevent burnout. This study aimed to answer the following research questions: (1) Is there a difference in dietetic interns’ and physician assistant interns’ fruit and vegetable intake? (2) Is there a difference in dietetic interns’ and physician assistant interns’ food, housing, and transportation security? (3) Is there a difference in dietetic interns’ and physician assistant interns’ barriers to healthy eating during an internship? (4) Is there a difference in dietetic interns’ and physician assistant interns’ empowerment for making healthy dietary choices during an internship?

We hypothesized that dietetic interns would have a higher intake of fruits and vegetables and higher empowerment for making health dietary choices during an internship.

## 2. Materials and Methods

This cross-sectional pilot study assessed a convenience sample of dietetic interns’ and physician assistant interns’ dietary intake, food security, barriers to healthy eating during an internship, and empowerment for making healthy dietary choices during an internship. Setting: Participants completed the online survey at the time and location of their choice. Recruitment and survey completion occurred in January and February 2023. Participants: Current dietetic interns and current physician assistant interns who were completing rotations, or had completed their rotation in the past six months, were eligible to participate. Individuals who did not meet these criteria were excluded from participating. Participant recruitment included forwarded emails from directors of accredited dietetics [13] and physician assistant [14] programs, and electronic fliers posted on the researchers’ social media accounts (Facebook, Instagram, and LinkedIn) along with well-known dietetics social media accounts. Interested interns followed a link or QR code included in the email or flier to access the anonymous, open, Qualtrics (Provo, UT, USA) [15] survey. All study procedures were reviewed and approved by the university Institutional Review Board; the study (Protocol #13090) was classified as exempt. A letter of information was included at the start of the survey as part of the consent process. Information was provided on the purpose of study, voluntary nature, study procedures, the estimated length of the study, and the option to skip any questions the participants did not feel comfortable answering. By continuing to the survey, individuals affirmed that they wished to participate in the study.

Variables: The survey assessed self-reported fruit and vegetable intake, food security, barriers to healthy eating during an internship, and perceived empowerment for making healthy dietary choices during an internship. No variables that were potential confounders were included. The survey consisted of eight pages, each containing approximately six questions. Participants could choose not to answer any questions, but once they submitted their answers, they could not go back and change them. All participants answered questions in the same order and there was no randomization of questions. Adaptive questioning was used to ensure that questions were only provided to participants if they were applicable based on their previous responses. Data Sources: The survey included questions that asked how many servings per day of fruits and vegetables, respectively, were eaten during the internship; the options included 0 servings per day, 1–2 servings per day, 3–4 servings per day, 5–6 servings per day, and more than 6 servings per day. This is similar to the format used in a food frequency questionnaire (FFQ), a tool that is often used in nutrition studies [16]. The survey also included questions from the Six-Item Food Security Scale developed by the National Center for Health Statistics [17], the Accountable Health Communities Screening Tool [18], and questions from the Diabetes Empowerment Scale [19] that were adapted to be specific to healthy eating during an internship. The survey also included questions that addressed barriers to healthy eating during an internship that were identified in previous research [1]; a 5-point Likert scale was used ranging from strongly agree to strongly disagree. Bias: To address potential sources of bias, the researchers pilot-tested the survey questions to establish face validity [20], avoided the use of leading questions, and considered cultural variations when designing the study. Researchers also assessed and ensured that the online survey was functional and useable prior to data collection. Study Size: Sample size calculations were not conducted due to the absence of existing data on interns’ empowerment for healthy eating during an internship. This study was considered a pilot study; therefore, 100 participants in each group (100 dietetic interns and 100 physician assistant interns) were the target sample size, which is in line with previous cross-sectional pilot studies [10,21].

Quantitative Variables: Likert scale responses were converted to numbers (1–5) for analysis [19]. Statistical Methods: Independent *t*-tests and chi-square distributions were conducted to assess differences in physician assistant interns’ and dietetic interns’ responses. Chronbach’s Alpha was used to assess the internal consistency of scales. An IBM Statistical Package for Social Sciences (IBM SPSS version 29; Chicago, IL, USA) [22] was used for all statistical analyses and the significance was set at *p* < 0.05. Because participants were not required to answer every question, there was some variation in the *n* values for each question. Only the responses given were included in the analysis; no imputations were conducted.

After completing the survey, all participants were directed to a link to provide contact information to enter a drawing for a chance to win a $15 Amazon electronic gift card. Participant responses were stored securely in a password-protected, encrypted folder on box.com. Participant contact information was stored in a separate folder on box.com. To minimize the risk of duplicate responses, survey settings were selected to prevent the survey from being completed twice from the same computer and internet browser. If a participant re-started the survey within one week of their original survey attempt, they were directed to where they left off in their initial attempt.

## 3. Results

### 3.1. Participant Characteristics

**Figure 1:**
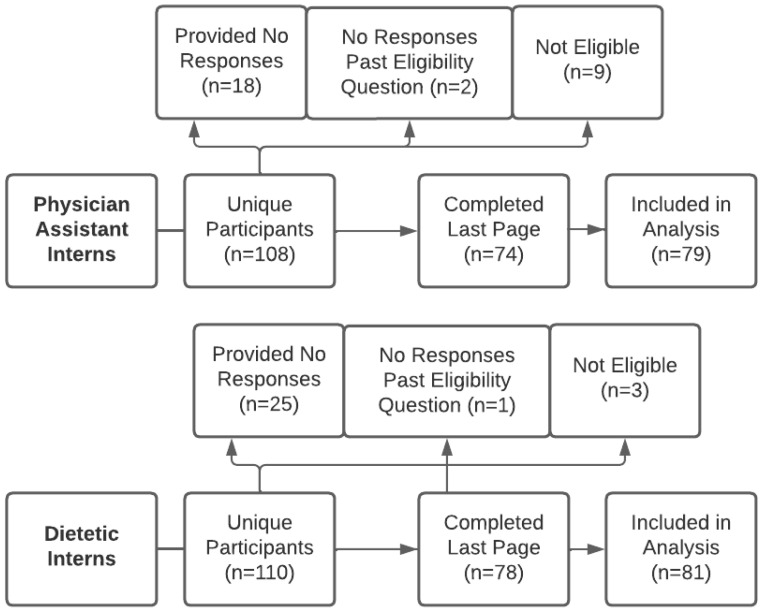
Survey completion flowchart.

The study’s recruitment methods make it unclear how many eligible individuals there were. A total of two hundred and eighteen unique participants accessed the survey. The participation rate (number of unique individuals who accessed the survey/number of unique individuals who agreed to participate) was 82%, and the completion rate (number of participants who accessed the last page of the survey/number of participants who agreed to participate) was 70%. Because participants were permitted to skip any questions of the survey they did not feel comfortable answering, 160 (73%) were included in analysis. See Figure 1 for further information regarding the reasons for non-participation. The majority of participants in both groups were female and Caucasian, although the physician assistant intern population had greater ethnic diversity (see Table 1). The majority of participants from both groups were from the Northeastern region of the United States (US). Dietetic interns had higher participation than physician assistant interns in the Western region, while physician assistant interns had higher participation in the Southeastern and Southwestern regions (see Table 1). The results for the 31 outcome variables are described in the remainder of the Section 3.

### 3.2. Fruit and Vegetable Intake

Dietetic interns (*n* = 81) had a higher vegetable intake than physician assistant interns (*n* = 79) (2.57 ± 1.30 cups/day vs 1.99 ± 1.12 cups/day, *p* = 0.002); however, there were no differences when comparing dietetic interns’ (*n* = 80) and physician assistant interns’ (*n* = 79) fruit intake (1.83 ± 0.96 vs. 1.56 ± 1.09 cups/day; *p* = 0.10).

### 3.3. Food, Housing, and Transportation Security

While most dietetic interns had secure housing and transportation access, just over half of the physician assistant interns reported security in these areas (see Table 1). In addition, physician assistant interns reported higher rates of food insecurity than dietetic interns (Table 1). Over half of the respondents for both categories indicated that they were aware of food assistance programs and the Supplemental Nutrition Assistance Program (SNAP). More dietetic interns (*n* = 78) were aware of SNAP benefits for students than physician assistant interns (*n* = 76) (78.2% vs 59.2%, *p* = 0.011), although there were no differences in participant awareness of personal SNAP eligibility (50% vs 44.7%, *p* = 0.51).

### 3.4. Barriers to Healthy Eating during an Internship

The 15 questions regarding barriers to healthy eating during an internship had a high level of internal consistency (Cronbach’s Alpha = 0.81). There were no group differences when comparing the overall Barriers Score (Table 2). In terms of individual barriers, the highest rated influence on dietary intake was time in both populations (see Table 2). Dietetic interns reported a greater impact from mental fatigue than physician assistant interns, while physician assistant interns reported greater impact from interactions with peers during rotations (Table 2). The factors that differed the most between the groups were interaction with peers, preceptors, and patients, with all factors being higher among physician assistant interns (see Table 2).

### 3.5. Empowerment for Making Healthy Dietary Choices during an Internship

The internal consistency of the items included in the Empowerment Scale was high (Cronbach’s Alpha = 0.84). There were no group differences in the overall empowerment scores for healthy eating during an internship (Table 3). Dietetic interns had greater awareness of the parts of their dietary intake that they were dissatisfied with (question 1, *p* = 0.001), knew what helped them stay motivated to care for their body during their internship compared to physician assistant interns (question 7, *p* = 0.034), and knew enough about themselves to make the right choices for themselves regarding their food and nutrition compared to physician assistant interns (question 8, *p* = 0.032).

## 4. Discussion

### 4.1. Participant Characteristics

The sample population of dietetic interns resembled the US demographics of dietetic interns and practicing RDs in terms of gender (92.3% female vs. 87% female and 92% female, respectively), although variability existed in terms of race (74% Caucasian vs. 59.5% Caucasian and 90% Caucasian, respectively) [23,24]. Physician assistant interns’ demographics were also similar to the rates among interns and practicing Physician Assistants in the US in terms of gender (75.7% female vs. 77.8% and 70.1%, respectively) [25,26]. The population of physician assistant interns in this study was also more ethnically diverse than the intern population and population of practicing Physician Assistants in the US (52.7% Caucasian vs. 76.2% and 80.6%, respectively) [25,26]. Because of the proximal similarity to the US populations of interns, the results are likely generalizable to the national population.

### 4.2. Fruit and Vegetable Intake

The results indicated that although there was no difference in fruit intake, vegetable intake was higher among dietetic interns compared to physician assistant interns. A study of practicing Physician Assistants suggests that the majority consider their nutrition training to be lacking [27]. Previous research suggests that higher health literacy in college students is associated with improved health habits and increased fruit and vegetable intake [28]. This same concept may explain why dietetic interns had a higher vegetable intake. Dietetic coursework may increase dietetic intern awareness of dietary recommendations, which may increase the likelihood of consuming more vegetables.

### 4.3. Food, Housing, and Transportation Security

The results also indicated that physician assistant interns had a higher prevalence of food, housing, and transportation insecurity than dietetic interns. A scoping review by Nikolaus et al. reports that 10–75% of college students are food insecure. Although the food insecurity rates for both dietetic interns (32.9%) and physician assistant interns (53.5%) fell within this range, the rate for physician assistant students was on the higher end [29]. Current studies indicated that more research needs to be conducted on the factors that affect food insecurity among college students [30]. However, it is thought that the higher food literacy among physician assistant interns could play a role in helping them avoid food insecurity. Dietetic interns and physician assistant interns face a significant amount of financial pressure during the last phase of their healthcare training. Students with food insecurity tend to report more mental health concerns and lower academic achievement than their peers [3,31]. Those who are food insecure often report being from marginalized populations, having less family support, and requiring more financial assistance [32]. Creating and sustaining a diverse workforce remains an important aspect of healthcare professional education [33]. Factors that could reduce education attainment by marginalized persons have the potential to impact the diversity of the healthcare workforce. There were no group differences in the awareness of food assistance programs or awareness of personal eligibility for food assistance programs. Greater awareness of available resources could improve food security. A lack of housing and transportation were also found to be a greater barrier among physician assistant interns, which could contribute to the higher overall food insecurity. However, it is unknown whether housing insecurity was due to economic hardship or a reflection of the internship experience (e.g., living with family or friends during a rotation).

### 4.4. Barriers to Healthy Eating during an Internship

Similar to previous research among dietetic interns [1], time was the highest rated barrier to healthy eating during an internship for both physician assistant interns and dietetic interns. Notable differences include dietetic interns being more impacted by mental fatigue and additional responsibilities. In contrast, physician assistant interns indicated a greater impact on dietary choices from rotation from patient influence, peer influence, and preceptor influence.

### 4.5. Empowerment for Making Healthy Dietary Choices during an Internship

There was no significant difference in empowerment for healthy eating during an internship when comparing physician assistant interns and dietetic interns. Dietetic interns had higher scores for three empowerment scale items: “I know what parts of my diet I am dissatisfied with”, “I know what helps me stay motivated to care for my body”, and “I know enough about myself to make personalized nutrition choices”. This could be related to the higher nutrition and food literacy among dietetic interns [28], giving them increased awareness of their diet. Physician Assistants are often the first line of nutrition communication about health concerns in patients. A lack of nutrition knowledge and awareness has the potential to impact patient outcomes [34]. The fact that both groups had an average rating of 3.3/5 for “I can ask for support or help with food and/or nutrition challenges during my internship when I need it” suggests room for improvement in student support measures.

### 4.6. Strengths and Limitations

This study is strengthened by the fact that the study population appears to be similar to existing data on the gender representation of interns and practicing professionals in the US [23,24,25,26]; however, these results may not be generalizable to other populations. The present study yielded significant results; however, it is possible that existing correlations were not detected due to the small sample size. Future prospective studies with a larger sample size would allow for a greater understanding of the impact of an internship on a student’s dietary habits. Although the study questions were based on validated survey tools (e.g., The Six-Item Food Security Scale [17], the Accountable Health Communities Screening Tool [18]), the questions were modified to specifically refer to the period of time during an internship, but the validity among this population was not assessed. However, the Diabetes Empowerment Scale [19] questions that were modified to investigate empowerment for healthy eating during an internship (rather than empowerment related to diabetes care) had high internal consistency. This study was limited in that it did not comprehensively assess interns’ diet quality or their self-reported burnout. Additionally, the question regarding housing security may have been misinterpreted by participants. The question asked if the participants had housing at this time but did not specify whether the participants had permanent housing or were temporarily staying with friends or family while completing a rotation.

## 5. Conclusions

Healthy eating and access to food are critical concerns for all individuals. Dietetic interns had a higher vegetable intake than physician assistant interns. The lack of nutrition-related knowledge in physician assistant interns may lead to both poor nutrition-related behaviors long term as well as a lack of ability to provide accurate nutrition-related education to the patients they serve. As Physician Assistants are often the first line of nutrition communication about health concerns in patients, increased knowledge and awareness of nutrition interventions and a willingness to include nutrition experts in patient care is needed to support positive patient outcomes. The results of this study demonstrate that food insecurity is a concern for dietetic and physician assistant interns, although the rates of food, housing, and transportation insecurity were higher among physician assistant interns. Future research could explore the correlation between food insecurity during internships and dietary practices as a healthcare provider. The ramifications of food insecurity could impact other goals related to healthcare professions, including the ability to prevent burnout and sustain a diverse pool of practitioners. The known impacts of food insecurity and poor nutrition are adequate to encourage program directors and higher education institutions to address nutrition and financial needs among future healthcare professionals. Both dietetic interns and physician assistant interns reported that finances and time were barriers to healthy eating during an internship. The barrier of mental fatigue was rated more highly by dietetic interns, while interactions with preceptors, peers, and patients were rated more highly by physician assistant interns. The interns in this survey reported high overall empowerment for healthy eating during their internship, and there were no differences between groups. However, physician assistant interns had lower overall confidence in their knowledge of what changes may be needed in their own diets. Despite high overall empowerment scores, addressing concerns with food insecurity, time, mental fatigue, and interns’ empowerment to ask for help when they need it could lead to increases in overall empowerment for all interns. Empowering students may increase the likelihood that they will be empowered healthcare providers. Addressing the unique concerns of dietetic interns and physician assistant interns during their internship may benefit interns during their training as well as develop practices that support the overall wellbeing of these healthcare professionals, leading to better patient interactions and outcomes.

## Figures and Tables

**Table 1:** Physician assistant (PA) intern and dietetic intern characteristics.

	PA * Intern (*n*)	Dietetic Intern (*n*)	PA Interns *n* (%)	Dietetic Interns *n* (%)	*p*-Value
Gender	74	78			0.015
Male			17 (23%)	5 (6.4%)	
Female			56 (75.7%)	72 (92.3%)	
Other			1 (1.4%)	0 (0%)	
Prefer not to say			0 (0%)	1 (1.3%)	
Age	74	78	26.01 ± 3.72	27.92 ± 9.61	0.11
Ethnicity	74	78			<0.001
American Indian or Alaska Native			1 (1.4%)	0 (0%)	
Asian			9 (12.2%)	3 (3.8%)	
Asian, Black or African American			0 (0%)	1 (1.3%)	
Asian, Caucasian			1 (1.4%)	2 (2.6%)	
Black or African American, Caucasian			0 (0%)	1 (1.3%)	
Caucasian			39 (52.7%)	58 (74.4%)	
Hispanic			3 (4.1%)	10 (12.8%)	
Hispanic, Caucasian			1 (1.4%)	2 (2.6%)	
Native Hawaiian or other Pacific Islander			2 (2.7%)	0 (0%)	
Region	72	78			0.042
Midwest			8 (11.1%)	11 (14.1%)	
Northeast			21 (29.2%)	23 (29.5%)	
Southeast			17 (23.6%)	13 (16.7%)	
Southwest			17 (23.6%)	7 (9.0%)	
West			8 (11.1%)	22 (28.2%)	
West, Midwest			1 (1.4%)	0 (0%)	
West, Northeast			0 (0%)	1 (1.3%)	
West, Southeast			0 (0%)	1 (1.3%)	
Housing Security	76	78			<0.001
I have housing			44 (54.9%)	72 (92.3%)	
I do not have housing			26 (34.2%)	1 (1.3%)	
I have housing today, but am worried about losing housing in the future			6 (7.9%)	5 (6.4%)	
Transportation Security	76	78			<0.001
No			42 (55.2%)	71 (91.0%)	
Yes, it has kept me from medical appointments or getting medications			12 (15.8%)	1 (1.3%)	
Yes, it has kept me from non-medical meetings, appointments, work, or getting things that I need			15 (19.7%)	4 (5.1%)	
Food Security	71	76			0.040
Food Secure			33 (46.5%)	51 (67.1%)	
Low Food Security			17 (23.9%)	12 (15.8%)	
Very Low Food Security			21 (29.6%)	13 (17.1%)	

Data analysis was conducted using a Chi-Square test for gender, ethnicity, region, housing security, transportation security, and food security; an independent *t*-test was used for age. * Participants were not required to answer all questions; therefore, the *n* values vary.

**Table 2:** Mean impact (on a scale from 1 to 5) of barriers on healthy eating among physician assistant (PA) interns’ and dietetic interns’ dietary intake during an internship.

	PA * Interns	Dietetic Interns	PA Interns	Dietetic Interns	*p*-Value
	(*n*)	(*ni*)	Mean ± SD	Mean ± SD	
Overall Barriers Score **	74	78	53.27 ± 9.00	51.40 ± 9.29	0.209
Time	76	79	4.45 ± 0.82	4.52 ± 0.88	0.601
Finances	76	79	4.14 ± 1.13	3.96 ± 1.28	0.347
Food Access During Rotations	76	79	3.79 ± 1.08	3.39 ± 1.45	0.055
Food Access Outside Rotations	76	79	3.03 ± 1.37	2.76 ± 1.42	0.235
Physical Fatigue	75	79	3.91 ± 1.04	4.10 ± 1.02	0.243
Mental Fatigue	76	79	4.01 ± 1.07	4.43 ± 0.76	0.006
Additional Responsibilities	76	79	3.88 ± 1.01	4.19 ± 1.03	0.061
Health Goals	76	79	3.68 ± 1.04	3.78 ± 0.97	0.519
Preferences	76	79	3.62 ± 1.12	3.80 ± 1.13	0.322
Living Situation	76	79	3.39 ± 1.24	3.52 ± 1.32	0.548
Rotation Culture	76	79	3.47 ± 1.12	3.16 ± 1.34	0.133
Interactions with Peers During Rotation	75	78	3.09 ± 1.18	2.51 ± 1.15	0.002
Interactions with Peers Outside of Rotation	75	79	2.97 ± 1.19	2.58 ± 1.19	0.041
Interactions with Patients	75	79	2.76 ± 1.26	2.20 ± 1.22	0.006
Interactions with Preceptors	75	79	3.11 ± 1.20	2.46 ± 1.28	0.001

Data analysis was conducted using independent *t*-tests. * Participants were not required to answer all questions; therefore, the *n* values vary. ** Maximum score of 40.

**Table 3:** Physician assistant (PA) interns’ (*n* = 76) * and dietetic interns’ (*n* = 80) empowerment for healthy eating during an internship.

	PA Interns Mean ± SD	Dietetic Interns Mean ± SD	*p*-Value
Overall Empowerment Score **	30.29 ± 5.74	31.54 ± 5.22	0.157
In general I believe that I: ***			
1. Know what parts of my dietary intake or nutrition during my internship I am dissatisfied with.	4.07 ± 0.75	4.48 ± 0.81	0.001
2. Am able to turn my food and/or nutrition-related goals into workable plans during my internship.	3.49 ± 1.14	3.76 ± 0.92	0.099
3. Can try out different ways of overcoming barriers to my food and/or nutrition-related goals during my internship.	3.91 ± 1.01	3.71 ± 0.94	0.213
4. Can find ways to feel better about having struggles with food and/or nutrition during my internship.	3.79 ± 1.00	3.83 ± 0.94	0.819
5. Know the positive ways I cope with stress related to food and/or nutrition during my internship.	3.71 ± 0.96	3.81 ± 1.13	0.545
6. Can ask for support or help with food and/or nutrition challenges during my internship when I need it.	3.33 ± 1.27	3.31 ± 1.23	0.935
7. Know what helps me stay motivated to care for my body through food and/or nutrition during internship.	3.87 ± 1.09	4.21 ± 0.92	0.034
8. Know enough about myself as a person to make food and/or nutrition-related choices during my internship that are right for me.	4.13 ± 0.90	4.43 ± 0.79	0.032

Data analysis was conducted using independent *t*-tests. * Participants were not required to answer all questions; therefore, the *n* values vary. ** Maximum score of 40. *** Maximum score of 5.

## Data Availability

The original contributions presented in the study are included in the article, further inquiries can be directed to the corresponding author.
